# Efficacy of Selective Serotonin Reuptake Inhibitors for the Treatment of Chronic Pain and Comorbid Depression in Individuals With Fibromyalgia: A Systematic Review and Meta-Analysis of Randomized Controlled Trials

**DOI:** 10.7759/cureus.102998

**Published:** 2026-02-04

**Authors:** Salman Alfawaz, Zakiyah Aljeshi, Jehad Aldandan, Muath Alharthi, Mohammed Alnefaie, Nada Babelli, Nouf Alshehri, Shaden Alsenaidi, Wael Albalawi, Ghadah Alghamdi, Sarah Alsufyani, Aziz Alfeeli

**Affiliations:** 1 Physical Medicine and Rehabilitation, Ministry of Health - Kuwait, Kuwait City, KWT; 2 College of Medicine, Alfaisal University, Riyadh, SAU; 3 College of Medicine, King Faisal University, Hofuf, SAU; 4 College of Medicine, Taif University, Taif, SAU; 5 College of Medicine, University of Jeddah, Jeddah, SAU; 6 Neurology, King Fahad Medical City, Riyadh, SAU; 7 College of Medicine, University of Tabuk, Tabuk, SAU; 8 College of Medicine, Almaarefa University, Riyadh, SAU; 9 College of Pharmacy, Qassim University, Buraydah, SAU; 10 College of Medicine, King Abdulaziz University, Jeddah, SAU; 11 Psychology, Washington Adventist University, Takoma Park, USA; 12 Physical Medicine and Rehabilitation, Al-Amiri Hospital, Kuwait City, KWT

**Keywords:** chronic widespread pain, citalopram, escitalopram, fibromyalgia, fibromyalgia syndrome, fluoxetine, paroxetine, selective serotonin reuptake inhibitor, sertraline, ssri

## Abstract

Fibromyalgia causes symptoms like chronic widespread pain, fatigue, and cognitive difficulties, often alongside depression and chronic pain, complicating treatment. While selective serotonin reuptake inhibitors (SSRIs) are prescribed for fibromyalgia-related depression and pain, their effectiveness is unclear. This systematic review and meta-analysis evaluated SSRI efficacy in fibromyalgia patients with comorbid depression and chronic pain.

This systematic review was registered with the International Prospective Register of Systematic Reviews (PROSPERO) and conducted in accordance with the Preferred Reporting Items for Systematic Reviews and Meta-Analyses (PRISMA) 2020 guidelines. We searched PubMed, Cochrane Central Register of Controlled Trials (CENTRAL), Web of Science, Ovid, and Google Scholar. We included randomized controlled trials (RCTs) using formal fibromyalgia criteria and reporting depression or pain outcomes. Non-RCTs, studies with non-adults, or non-SSRI comparisons without isolating SSRI effects were excluded. The Cochrane risk-of-bias tool for randomized trials (RoB-2) was used to assess the risk of bias in this study. Four reviewers independently screened titles and abstracts; four assessed full-texts with disagreements resolved by a senior reviewer. Outcome data were converted to mean change scores from baseline where not directly reported. The standardized mean difference (SMD) was used for the meta-analysis to pool data across different scales.

Nine RCTs (n = 461 participants, predominantly female, mean ages across studies ranged from 32.7 to 52.9 years) were analyzed. Compared to placebo, SSRIs significantly reduced pain (eight RCTs, n = 421; SMD -0.53, 95% confidence interval (CI): -0.92 to -0.14, p = 0.007) and improved depression (six RCTs, n = 265; SMD -0.67, 95% CI: -1.14 to -0.20, p = 0.005). SSRIs also significantly improved quality of life (QOL) versus placebo (five RCTs, n = 301; SMD -0.30, 95% CI: -0.55 to -0.04, p = 0.02). Compared to other non-pharmacological interventions (acupuncture and aerobic exercise), SSRIs showed non-significant improvement in depression (two RCTs, n = 70; SMD -0.39, 95% CI: -1.32 to 0.54, p = 0.41). Common side effects included gastrointestinal issues, dry mouth, sedation, sexual dysfunction, and headaches; SSRI groups reported more adverse events and higher dropout rates.

SSRIs showed statistically significant benefits for pain, depression, and QOL in fibromyalgia compared to placebo. However, the overall evidence quality was found to be very low to low due to heterogeneity and risk of bias. Future RCT designs should adhere to a strict methodology in order to strengthen the available evidence on the efficacy of SSRIs in treating fibromyalgia.

## Introduction and background

Fibromyalgia is a chronic disorder characterized by a range of symptoms of musculoskeletal pain, fatigue, sleep disturbances, and cognitive impairments. It affects approximately 2-4% of the global population and disproportionately impacts women [1]. Depression is a common comorbidity, with up to 60-65% of individuals with fibromyalgia experiencing major depressive disorder during their lifetime [2,3]. The coexistence of chronic pain and depression significantly worsens functional outcomes and quality of life (QOL), posing substantial challenges to effective management [2,3].

Selective serotonin reuptake inhibitors (SSRIs) are widely used for the treatment of major depressive disorder [4] and have also been considered for addressing chronic pain syndromes, including fibromyalgia [5]. Their proposed analgesic effect may stem from the modulation of central pain pathways through serotonergic mechanisms [6]. Despite their frequent use in clinical practice, evidence on the efficacy of SSRIs in treating both pain and mood symptoms in fibromyalgia remains inconsistent across studies, and their role in improving broader outcomes such as QOL and daily functioning is not well established [5,6].

A systematic review by Walitt et al. (2015) evaluated the efficacy of SSRIs in fibromyalgia patients. The review concluded that there was no unbiased evidence that SSRIs were more effective than placebo in treating pain, fatigue, and sleep problems. However, SSRIs might be considered for treating depression in people with fibromyalgia [5].

On the other hand, a meta-analysis by Häuser et al. (2012) assessing the role of antidepressants in managing fibromyalgia found that while antidepressants, including SSRIs, can have positive effects on pain, sleep, and overall well-being, their effect size was modest. The authors noted that the analgesic effects of antidepressants were independent of their antidepressant effects, suggesting a distinct mechanism of action in pain modulation [6].

Patkar et al. (2007) conducted a randomized controlled trial (RCT) that investigated the efficacy of paroxetine, an SSRI, in fibromyalgia patients without current major depression or anxiety disorders. The study found that paroxetine was significantly superior to placebo in reducing the Fibromyalgia Impact Questionnaire (FIQ) total score and improving Clinical Global Impression ratings. However, the improvements in other secondary outcome measures between the two groups were not statistically significant [7].

Although several other RCTs have evaluated the efficacy of SSRIs in fibromyalgia, the findings remain inconsistent. Some studies reported modest benefits in pain reduction, mood improvement, and functional status, while others found limited or no significant effects beyond those of the placebo. The variability in patient selection criteria, intervention protocols, SSRIs assessed, outcome measures, and study quality contributes to this lack of consensus in the literature [5,7].

This study aimed to assess the efficacy of SSRIs in reducing chronic pain and improving comorbid depression, QOL, and functional status in addition to evaluating the safety and tolerability of SSRIs in adults with fibromyalgia.

## Review

Methods

Registration and Study Design

We registered our study protocol in the International Prospective Register of Systematic Reviews (PROSPERO) (ID: CRD420251031894) [8]. Furthermore, we followed the Preferred Reporting Items for Systematic Reviews and Meta-Analyses (PRISMA) guidelines when conducting the study [9].

Inclusion and Exclusion Criteria

Our systematic review included studies published in English that focused on adults over 18 years old diagnosed with fibromyalgia according to established diagnostic criteria, such as the American College of Rheumatology (ACR) criteria [10]. The inclusion criteria specified that participants must have comorbid chronic pain and/or depression. Only studies that administered any SSRI, including fluoxetine, sertraline, paroxetine, citalopram, or escitalopram, were considered. Additionally, all studies needed to include a control group receiving either a placebo, no treatment, or other active pharmacological or non-pharmacological interventions. The outcomes reported had to be related to patient-reported or clinician-assessed measures of pain intensity, depressive symptoms, QOL, functional status, and adverse events. Only RCTs published in peer-reviewed journals were eligible for inclusion. There were no restrictions on the publication date, and all studies published up to the date of the search were included.

Exclusion criteria comprised studies involving pediatric populations or patients under 18 years old, patients without a formal fibromyalgia diagnosis, and those utilizing non-SSRI antidepressants, such as serotonin-norepinephrine reuptake inhibitors (SNRIs), tricyclic antidepressants (TCAs), and monoamine oxidase inhibitors (MAOIs), or SSRIs in combination with other pharmacologic agents without isolated analysis. Studies that did not include a control or comparison group or that did not report pain or depression outcomes as primary or secondary outcomes were also excluded. Additionally, non-RCT studies, observational studies, case reports, qualitative studies, protocols, conference abstracts, and non-peer-reviewed literature were not considered. The inclusion and exclusion criteria are summarized in Table 1. 

**Table 1:** Inclusion and exclusion criteria SSRI: selective serotonin reuptake inhibitor; QOL: quality of life; RCT: randomized controlled trial; SNRI: serotonin-norepinephrine reuptake inhibitor; TCAs: tricyclic antidepressant; MAOI: monoamine oxidase inhibitor; ACR: American College of Rheumatology

Domain	Inclusion Criteria	Exclusion Criteria
Population	Adults (≥18 years) diagnosed with fibromyalgia according to established diagnostic criteria (e.g., ACR criteria), with comorbid chronic pain and/or depression	Pediatric populations; patients without a formal diagnosis of fibromyalgia; patients without co-occurring depression
Intervention	Administration of any SSRI (e.g., fluoxetine, sertraline, paroxetine, citalopram, escitalopram)	Use of non-SSRI antidepressants (e.g., SNRIs, TCAs, MAOIs) or SSRIs used in combination with other pharmacologic agents without isolated analysis
Comparison	Placebo, no treatment, or other active pharmacological or non-pharmacological interventions	Studies lacking a control or comparison group
Outcomes	Patient-reported or clinician-assessed outcomes related to pain intensity, depressive symptoms, QOL, functional status, and adverse events	Studies that did not report pain or depression outcomes as primary or secondary outcomes
Study Design	RCTs published in peer-reviewed journals	Non-RCTs, observational studies, case reports, qualitative studies, protocols, conference abstracts, and non-peer-reviewed literature

Search Strategy

We conducted comprehensive research using multiple databases, including PubMed, Google Scholar, Cochrane Central Register of Controlled Trials (CENTRAL), Web of Science, and Ovid. The literature search spanned from inception to April 14, 2025, without any limitations. We used the following search strategy to identify similar studies: fibromyalgia OR "fibromyalgia syndrome" OR "chronic widespread pain" AND ("selective serotonin reuptake inhibitor" OR SSRI OR fluoxetine OR sertraline OR paroxetine OR citalopram OR escitalopram).

Screening and Selection of Studies

**Figure 1:**
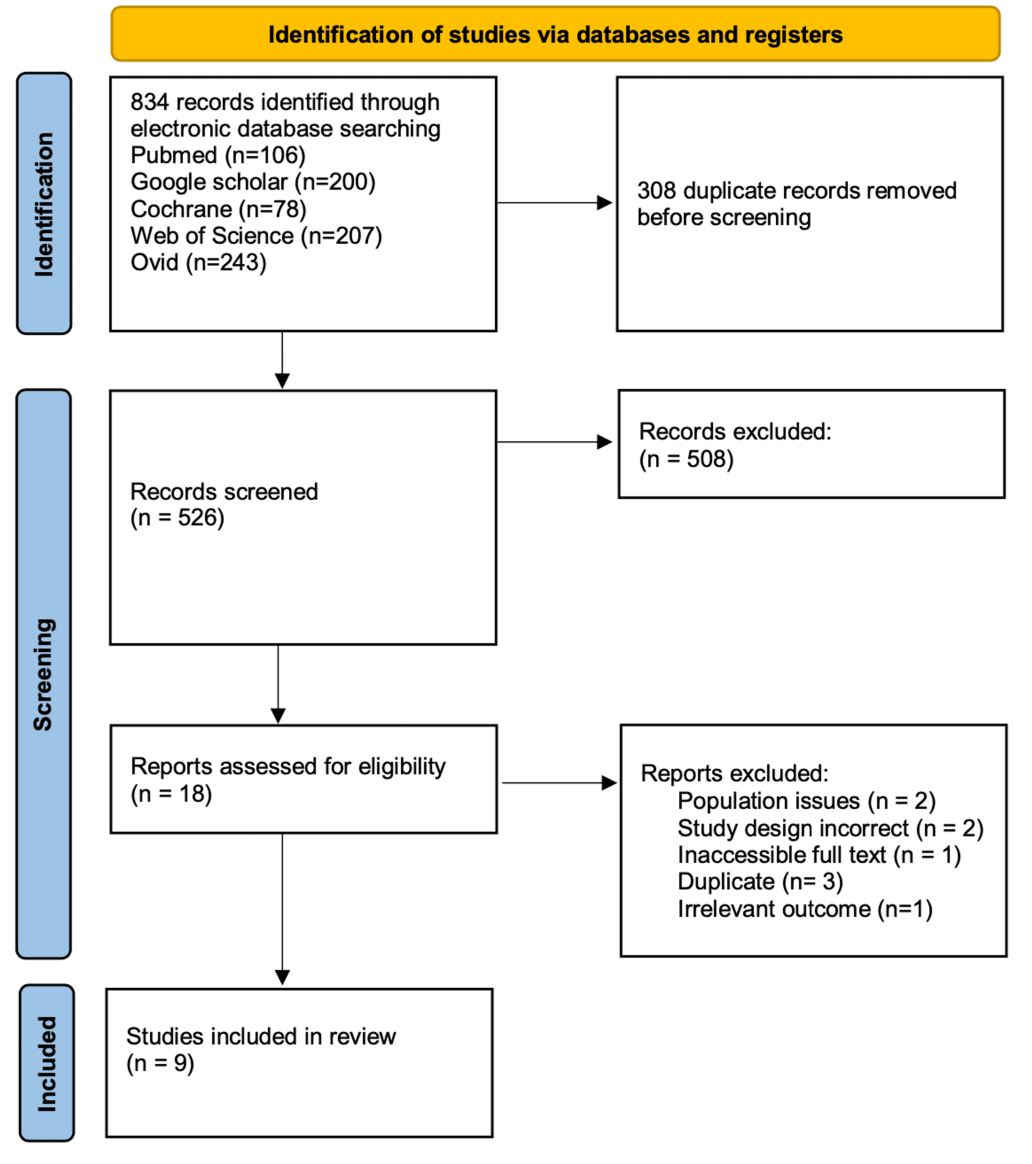
PRISMA flow diagram PRISMA: Preferred Reporting Items for Systematic Reviews and Meta-Analyses

After downloading the identified studies from various electronic databases (834 studies were downloaded, and 308 were removed as duplicates), we transferred them to the Rayyan website for screening by title and abstract (526 studies remained for screening) [11]. Four individuals independently reviewed 100% of the articles and included the potentially eligible articles for further review by full-text screening (18 out of 526 articles were included). Finally, another four individuals independently reviewed the articles, assessing their full texts for eligibility (nine out of 18 studies were ultimately included in our systematic review). Any disagreements among authors were resolved by consulting a senior reviewer. We documented the screening process, including reasons for exclusion, using a PRISMA flow diagram to provide a clear overview of the articles identified (Figure 1).

The primary objective of this systematic review was to assess outcomes associated with chronic pain reduction in adults with fibromyalgia.

Data Extraction

After full-text screening, data were extracted by two independent groups. Each group consisted of two authors: one author extracted data from the assigned articles, and the other verified the extraction to ensure completeness and accuracy.

We used a standardized extraction form to collect data on study design, participant characteristics, intervention and control details (type, dose, duration), outcome measures (e.g., pain, depression, QOL), adverse events, and dropout rates.

Risk of Bias and Quality Assessment

To evaluate the quality of the RCTs, the included studies were assessed using the Cochrane risk-of-bias tool for randomized trials (RoB-2) [12]. This tool encompasses bias resulting from five domains: bias due to randomization, bias due to deviations from intended interventions, bias secondary to missing outcome data, bias due to outcome measurement, and bias in the selection of the reported result. An algorithm is then applied for each domain to make judgments, which can be classified as 'low' (indicating low risk of bias in all domains), 'some concerns' (suggesting concerns in at least one domain), or 'high' (indicating a high risk in one or more domains or concerns in multiple domains).

Statistical Analysis

All outcome data were converted to mean change scores from baseline. When standard deviations (SDs) were not reported directly, they were derived using a validated calculator as recommended in the Cochrane Handbook [13]. Due to variations in outcome measurement tools across studies (e.g., different pain or depression scales), we calculated standardized mean differences (SMDs) with 95% confidence intervals (CIs) to allow pooling across different scales.

A random-effects model was applied to account for expected heterogeneity among trials. Statistical heterogeneity was assessed using the chi-square test (p <0.10 indicating significance) and quantified with the I² statistic, with values of 25%, 50%, and 75% representing low, moderate, and high heterogeneity, respectively. All analyses were performed using Review Manager (RevMan) 5.4.1 (The Cochrane Collaboration, London, England, UK), and a two-sided p <0.05 was considered statistically significant.

Results

Results of the Search

The literature search was conducted across the Cochrane Library, Ovid platforms, Web of Science, PubMed, and Google Scholar, with a search range from inception to April 14, 2025. The search resulted in 834 potentially relevant citations, of which 308 were duplicate records. As a result, 526 unique citations underwent initial screening based on titles and abstracts. The majority of references were excluded at this stage primarily for the following criteria: involvement of pediatric populations (<18 years old) or patients without a formal fibromyalgia diagnosis; investigation of non-SSRI antidepressants (e.g., SNRIs, TCAs, MAOIs) or SSRIs combined with other pharmacologic agents where isolated effects could not be determined; absence of a control/comparison group or failure to report pain or depression outcomes as primary or secondary measures; and non-RCT study designs (including observational studies, case reports, qualitative studies, protocols, conference abstracts, and non-peer-reviewed literature). This process resulted in the selection of 18 articles for full-text screening. Following a detailed evaluation of these full texts, nine studies were excluded (specific reasons for exclusion at the full-text stage are provided in the PRISMA flow diagram (Figure 1)).

Consequently, nine RCTs met the pre-defined inclusion criteria and were included in the final analysis. The characteristics of the included studies are provided in Table 2.

**Table 2:** Characteristics of the included RCTs evaluating SSRIs in fibromyalgia SSRI: selective serotonin reuptake inhibitor; SD: standard deviation; RCT: randomized controlled trial; wk: weeks; NR: not reported; TENS: transcutaneous electrical nerve stimulation; a: SSRI group; b: aerobic exercise group; c: placebo TENS group; N/A: not applicable due to crossover study design; CR: controlled release

First Author / Year	Country	Study Design	Sample Size (SSRI / Control / Total)	Age Mean ± SD (SSRI / Control)	Gender (% Female)	SSRI Type and Dosage	Duration	Control Type and Dosage
Patkar, 2007 [7]	United States	RCT	58 / 58 / 116	47.9 ± 9.1 / 49.1 ± 11.2	94%	Paroxetine CR up to 62.5 mg	12 wk	Placebo
Goldenberg, 1996 [14]	United States	RCT (crossover)	31 (N/A)	Overall: 43.2 ± 9.1	90%	Fluoxetine 20 mg	6 wk	Crossover: Placebo, Amitriptyline, Amitriptyline + Fluoxetine
Nørregaard, 1995 [15]	Denmark	RCT	21 / 21 / 42	48 ± 9 / 50 ± 9	NR	Citalopram 20-40 mg	8 wk	Placebo
Hadianfard, 2012 [16]	Iran	RCT (single-blind)	15 / 15 / 30	44.2 ± 10.8 / 43.9 ± 7.9	100%	Fluoxetine 20 mg	8 wk	Acupuncture
Anderberg, 2000 [17]	Sweden	RCT	21 / 19 / 40	Overall: 48.6 ± 7.5	100%	Citalopram 20-40 mg	16 wk	Placebo
Sencan, 2004 [18]	Turkey	RCT	20 / 40 / 60	32.6 ± 9.4 (a) / 35.4 ± 9.62 (b) / 35.55 ± 7.86 (c)	100%	Paroxetine 20 mg	6 wk	Aerobic Exercise / Placebo TENS
Arnold, 2002 [19]	United States	RCT	30 / 30 / 60	46 ± 11 / 46 ± 12	100%	Fluoxetine up to 60 mg	12 wk	Placebo
Wolfe, 1994 [20]	United States	RCT	21 / 21 / 42	48 ± 10.1 / 52.9 ± 11.3	100%	Fluoxetine 20 mg	6 wk	Placebo
Giordano, 1999 [21]	Italy	RCT (single-blind)	20 / 20 / 40	Overall: 31 ± 7.2	100%	Paroxetine 20 mg	12 wk	Placebo

Included Studies

*S*tudy designs: This systematic review included nine RCTs published between 1994 and 2012. The nine trials comprised one crossover study [14] and eight parallel-group designs [7,15-21]. There were two single-blind studies [16,21] and one three-arm trial that compared paroxetine, aerobic exercise, and placebo [18]. All the other trials used double-blind designs. A total of 461 participants were randomized across the studies. The drug-specific allocations were as follows: citalopram (n = 42), fluoxetine (n = 97), paroxetine (n = 98), and placebo (n = 255). The crossover trial [14] involved 31 participants who were exposed to each of the following: amitriptyline, fluoxetine/amitriptyline, fluoxetine, and placebo. Sample sizes ranged from 30 to 116 participants. 

Funding sources: Pharmaceutical industry funding was reported in five studies: GlaxoSmithKline (GSK) [7], Lilly Research Laboratories [19,20], and H. Lundbeck A/S [15,17]. Public or institutional grants supported three trials, including the Lot Page Fund (Newton-Wellesley Hospital) [14], Shiraz University of Medical Sciences [16], the Söderström Königska Foundation, the Swedish Association of Physicians, the Märta and Nicke Nasvell Foundation, the Swedish Health Insurance System, the Uppsala County Council and 'Förenade Liv' Mutual Group Life Insurance Company, Stockholm, Sweden, and the Swedish Medical Research Council [17].

Baseline characteristics: Study populations were predominantly female (100% in six trials [16-21]), while two other trials reported female representation of 90.3% [14] and 94% [7]. All participants were adults (aged ≥18 years) diagnosed according to ACR criteria. Inflammatory rheumatic diseases were explicitly excluded in six studies [15,16,18-21], while three studies failed to adequately confirm their exclusions [7,14,17]. Major depressive symptoms were formally excluded in six trials [7,15-19], with three lacking documentation [14,20,21]. The participants' ages across the eight studies ranged from 18 to 71 years. One study did not provide information on the age range [15]. The mean age of participants was 43.90 ± 6.64 years.

Study locations: Four of the nine studies were based in the United States [7,14,19,20], while the remaining studies originated from Denmark [15], Sweden [17], Iran [16], Italy [21], and Turkey [18].

Study durations: Interventions varied in length. The shortest duration was six weeks in three studies [14,18,20], followed by eight weeks in two studies [15,16]. Exceeding it were three studies spanning 12 weeks [7,19,21] and a single study lasting 16 weeks [17].

Interventions

SSRI regimens versus placebo: Citalopram was evaluated in two trials (20-40 mg/day [15,17]). Fluoxetine was assessed in three placebo-controlled trials implementing 20 mg/day as the dose: one utilized dose titration (initial 20 mg/day, increasing by 10-20 mg/week based on tolerance/response [19]), while the other two maintained a fixed 20 mg/day dose [14,20]. Paroxetine was investigated in three trials: one employed flexible dosing (initial 12.5 mg/day, titrated weekly to a maximum of 62.5 mg/day [7]), and two used fixed 20 mg/day dosing [18,21].

SSRI versus non-pharmacological interventions: Two trials evaluated SSRIs against non-placebo controls; the first compared fluoxetine 20 mg/day with acupuncture [16], and the other contrasted paroxetine 20 mg/day with aerobic exercise in a three-arm trial design [18].

SSRI versus other drugs: A single study compared the effect of an SSRI (fluoxetine) to a TCA (amitriptyline) in a crossover trial [14]. Since none of the other included studies compared SSRIs to other drugs, a meta-analysis of this intervention could not be conducted.

Outcomes

Pain relief outcomes: Pain outcomes were reported in eight studies [7,14,15,17-21] using heterogeneous instruments such as the Visual Analog Scale (VAS) scaled (0-100) in two studies [7,14], (0-10) in three studies [15,17,18], and (0-3) in one study [20], in addition to the Tender Point Score from (1-5) in a single study [21], and the McGill Pain Questionnaire (MPQ) (0-78) in another [19]. A single study reported the outcome of pain as a median and range [16] and thus could not be included in the meta-analysis of pain.

Depression outcomes: Depression was assessed in six placebo-controlled trials [14,15,17-20] and two non-pharmacological intervention studies [16,18]. In placebo-controlled trials, Beck's Depression Inventory (BDI) was utilized without a clear range in two of the studies [14,18] and with a range of (0-36) in two other studies [15,20], while the Montgomery Åsberg Depression Rating Scale (MADRS) (1-6) was used in one study [17], and the FIQ depression subscale (0-10) was used in another [19]. On the other hand, the non-pharmacological intervention trials measured their results using the BDI in one study [18] and the FIQ depression subscale in another [16] without providing specific ranges.

Although an additional study comparing SSRI to placebo utilized the BDI to report the depression baseline, a post-intervention value was not provided [7]. Moreover, a single study utilized the Hamilton Depression Rating Scale (HAM-D) and reported a lack of correlation between SSRI use and improvement in depression without providing numerical data [21]. Therefore, the aforementioned studies could not be included in the meta-analysis presented in this section.

QOL outcomes:* *QOL was measured in five studies [7,14,15,19,20], primarily with the FIQ: a full-scale range of 0-80 was used in one study [19], 0-100 in another [7], and an unspecified range in two studies [14,15]. The Health Assessment Questionnaire (HAQ) Functional Disability (0-3) tool was used in the remaining study [20].

Risk of Bias in Included Studies

The methodological quality assessment revealed some concerns in six studies [7,14-17,19] and a high risk in three studies [18,20,21]. A detailed summary is provided in Table 3.

**Table 3:** Risk of bias assessment using the RoB-2 tool D1-D5 represent the five domains of the RoB-2 tool [12]. D1: bias due to randomization; D2: bias due to deviations from intervention; D3: bias secondary to missing outcome data; D4: bias due to outcome measurement; D5: bias in the reported result selection; RoB-2: Cochrane risk-of-bias tool for randomized trials

First Author / Year	Study Type	D1	D2	D3	D4	D5	Overall Risk
Patkar, 2007 [7]	RCT	Low	Some Concerns	Low	Low	Some Concerns	Some Concerns
Goldenberg, 1996 [14]	RCT (crossover)	Low	Low	Some Concerns	Low	Low	Some Concerns
Nørregaard, 1995 [15]	RCT	Some Concerns	Low	Some Concerns	Low	Low	Some Concerns
Hadianfard, 2012 [16]	RCT (single-blind)	Low	Some Concerns	Low	Low	Low	Some Concerns
Anderberg, 2000 [17]	RCT	Low	Some Concerns	Some Concerns	Low	Some Concerns	Some Concerns
Sencan, 2004 [18]	RCT	Some Concerns	High Risk	High Risk	High Risk	Some Concerns	High Risk
Arnold, 2002 [19]	RCT	Low	Low	Some Concerns	Low	Some Concerns	Some Concerns
Wolfe, 1994 [20]	RCT	Some Concerns	Some Concerns	High Risk	High Risk	High Risk	High Risk
Giordano, 1999 [21]	RCT (single-blind)	Some Concerns	High Risk	High Risk	High Risk	Low	High Risk

Domain-Specific Assessments

Randomization and allocation concealment: The randomization method had some concerns in four studies: it was unclear in one study [18], while allocation concealment was inadequately described in three [15,20,21].

Blinding: Performance and detection bias risks were identified in six studies due to insufficient blinding procedures, with four studies displaying some concerns over blinding procedures [7,20], inability to double-blind [16], or intervention-related deviations [17], and two high-risk studies due to a lack of blinding methods [18,21].

Incomplete outcome data: One study reported no dropouts [16]. Concerns over high attrition (≥23%) were observed in four studies, raising some concern: 25.9% [7], 38.7% [14], 23% [15], and 38% [19]. Three studies exhibited disproportionately high-risk placebo group dropout rates [18,20,21].

Analysis methodology: Four studies used intention-to-treat (ITT) analysis [7,16,17,19], while one study additionally utilized last observation carried forward (LOCF) for ITT analysis [7]. Three studies were considered high risk as two studies lacked a clear method [18,21], while another single study inconsistently applied ITT and completed analyses [20].

Selective reporting: Six studies revealed concerns over potential reporting bias due to selective reporting of positive findings [7], lack of protocol transparency [17], no publicly available protocol/registration [18,19], absence of a prespecified analysis plan [20], and missing outcome data [21].

Effect of Interventions

Overall quality of evidence: The quality of evidence was rated as low for most outcomes due to substantial heterogeneity and a high risk of bias in studies [18,20,21], except for QOL outcomes, which demonstrated more substantial evidence.

**Figure 2:**
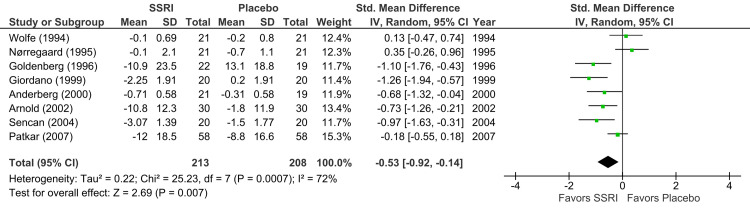
Forest plot showing the effect of SSRIs versus placebo on pain improvement Studies included (top to bottom), listed by first author and year: Wolfe (1994) [20], Nørregaard (1995) [15], Goldenberg (1996) [14], Giordano (1999) [21], Anderberg (2000) [17], Arnold (2002) [19], Sencan (2004) [18], Patkar (2007) [7]. SSRI: selective serotonin reuptake inhibitor; SD: standard deviation; CI: confidence interval; IV: inverse variance; Std.: standardized

Pain improvement (SSRI versus placebo): Eight RCTs (n = 421) demonstrated a statistically significant reduction in pain with SSRIs compared to placebo (p = 0.007), yielding a moderate effect size (SMD = -0.53; 95% CI = -0.92 to -0.14) (Figure 2). This result represents an appreciable clinical benefit; however, the quality of evidence was downgraded to low due to substantial heterogeneity (I² = 72%) [7,14,15,17-21].

**Figure 3:**
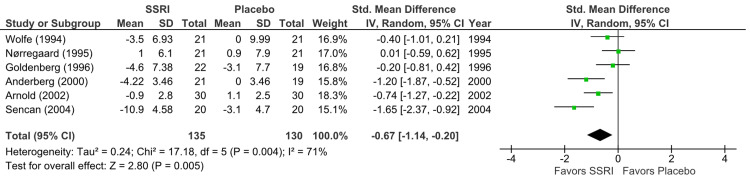
Forest plot showing the effect of SSRIs versus placebo on depression improvement Studies included (top to bottom), listed by first author and year: Wolfe (1994) [20], Nørregaard (1995) [15], Goldenberg (1996) [14], Anderberg (2000) [17], Arnold (2002) [19], Sencan (2004) [18]. SSRI: selective serotonin reuptake inhibitor; SD: standard deviation; CI: confidence interval; IV: inverse variance; Std.: standardized

Depression outcomes: Regarding SSRI versus placebo, six RCTs (n = 265) demonstrated statistically significant improvement in depression symptoms (p = 0.005) with a moderate effect size (SMD = -0.67; 95% CI = -1.14 to -0.20) (Figure 3). The quality of evidence was very low (I² = 71%) [14,15,17-20].

**Figure 4:**
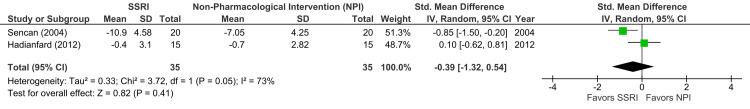
Forest plot showing the effect of SSRIs versus non-pharmacological intervention on depression improvement Studies included (top to bottom), listed by first author and year: Sencan (2004) [18], Hadianfard (2012) [16]. SSRI: selective serotonin reuptake inhibitor; SD: standard deviation; CI: confidence interval; IV: inverse variance; Std.: standardized

On the other hand, the analysis of SSRI versus non-pharmacological interventions in two RCTs (n = 70) [16,18] indicated a slight, non-significant improvement (p = 0.41, SMD -0.39; 95% CI = -1.32 to 0.54) (Figure 4) with low-quality evidence (I² = 73%).

**Figure 5:**
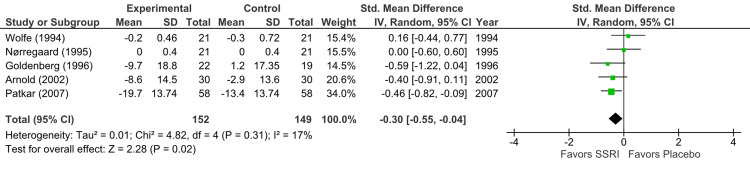
Forest plot showing the effect of SSRIs versus placebo on quality of life improvement Studies included (top to bottom), listed by first author and year: Wolfe (1994) [20], Nørregaard (1995) [15], Goldenberg (1996) [14], Arnold (2002) [19], Patkar (2007) [7]. SSRI: selective serotonin reuptake inhibitor; SD: standard deviation; CI: confidence interval; IV: inverse variance; Std.: standardized

QOL (SSRI versus placebo): Five RCTs (n = 301) reported a small but statistically significant improvement (p = 0.02) in QOL (SMD -0.30; 95% CI = -0.55 to -0.04) (Figure 5). The quality of evidence was high (I² = 17%), contrasting with the generally low evidence strength for other outcomes [7,14,15,19,20].

Heterogeneity Analysis

In placebo-controlled studies, heterogeneity was low for QOL outcomes (I² = 17%; Figure 5) but substantial for pain (I² = 72%; Figure 2) and depression (I² = 71%; Figure 3). Primary sources included variability in measurement scales and differential risk of bias. For non-pharmacological intervention studies, substantial heterogeneity was observed in depression outcomes (I² = 73%; Figure 4).

Adverse Effects

Adverse events associated with SSRIs were reported in all nine studies reviewed, which was consistent with our existing knowledge of their side effects. The most common issues were gastrointestinal, such as nausea (14-50%) and diarrhea (9-40%), especially with fluoxetine [19] and paroxetine [7,21]. Others included dry mouth (9-36%) and sedation (17-26%), along with sexual dysfunction (5-9%) and headaches (17-31%). SSRI groups reported more adverse events (15-65.5%) compared to placebos (7-58.6%), though placebo users also highlighted complaints such as headache and fatigue. Fluoxetine was tied to insomnia [19], paroxetine to dry mouth and sexual issues [18], and citalopram to nausea and dizziness [17]. Serious events were rare, with a few cases of renal infection and alcohol intoxication in paroxetine users [7] and severe nausea from citalopram leading to some quitting [17]. Dropout rates due to adverse events in the SSRI groups ranged from 12.5% to 38.7%, notably higher than placebo [14,17]. Some studies found significant differences in adverse events between SSRI and placebo, especially with paroxetine (Patkar et al., 2007; p <0.05) [7], but overall, many trials did not show major differences (Wolfe et al., 1994; p = 0.194) [20]. These findings match known SSRI patterns regarding tolerability, focusing on gastrointestinal and anticholinergic effects as key concerns in the treatment of fibromyalgia.

Discussion

Findings

Our systematic review and meta-analysis aimed to assess the efficacy of SSRIs in treating chronic pain and depression in adults with fibromyalgia. Our findings, based on nine RCTs involving 461 participants, indicate that SSRIs offer modest benefits when compared to placebo across all parameters, which include pain, depression, and QOL scales, yet highlight significant limitations in the existing evidence. Our analysis revealed that SSRIs were associated with a statistically significant, moderate reduction in pain compared to placebo across eight RCTs (p = 0.007; Figure 2). Similarly, a moderate and statistically significant improvement in depressive symptoms was observed in six RCTs (p = 0.005; Figure 3). Furthermore, five RCTs reported a small but statistically significant improvement in disease-specific QOL (p = 0.02; Figure 5). These results suggest that SSRIs may offer some therapeutic advantages for fibromyalgia patients.

A unique contribution of our study to the existing literature lies in its direct comparison of SSRIs against non-pharmacological interventions, namely acupuncture [16] and aerobic exercise [18], in the treatment of fibromyalgia. Our analysis of two RCTs investigating the depression outcome indicated a slight, non-significant improvement with SSRIs (p = 0.41), supported by low-quality evidence (I² = 73%). The limited sample size of these two studies (n = 70) restricts the statistical power to detect meaningful differences, if any exist (Figure 4). Unfortunately, we were unable to generate comparable results for the outcome of pain in one of the included studies due to an incompatibility in outcome reporting [16]. Similarly, QOL could not be assessed in the other study [18], which limits a comprehensive comparison across all key outcomes. Therefore, based on the limited sample size and low-quality evidence available from these comparisons, no definitive conclusion can be drawn regarding the superiority of SSRIs over non-pharmacological interventions for depression or any other outcome in fibromyalgia.

In examining the safety profile of SSRIs, although they appear to be tolerable, higher dropout rates may suggest the need for more adequate patient monitoring.

Comparison to Other Reviews

Generally, our findings align with the existing literature, which presents a mixed but often cautiously optimistic view on the efficacy of SSRIs in fibromyalgia. Our finding of a statistically significant reduction in pain with SSRIs is consistent with several previous meta-analyses. Walitt et al. (2015) found a small but statistically significant reduction in pain [5]. Similarly, Häuser et al. (2012) reported significant SMDs for pain with a small effect size [6]. Häuser et al. (2009) also found strong evidence for pain reduction, albeit with a small effect size [22]. O'Malley et al. (2000) described moderate symptomatic benefits for pain with antidepressants, including SSRIs [23]. However, Koechlin et al. (2021) highlighted significant placebo responses for pain, suggesting challenges in demonstrating the superior effects of drugs [24]. Our findings, while statistically significant, are qualified by low-quality evidence, echoing concerns raised by Walitt et al. (2015) regarding the very low quality of evidence for pain outcomes [5].

As for depression, our study found a statistically significant improvement in depression with SSRIs. Our findings are supported by Walitt et al. (2015) [5], who reported that SSRIs were statistically and clinically significantly superior to placebo in improving depression, albeit with a small effect size. Häuser et al. (2012) and Häuser et al. (2009) also noted significant, small effect sizes for depression improvement with SSRIs [6,22]. Üçeyler et al. (2008) indicated that 70% of studies reported improvement in depressiveness with SSRIs [25]. On the other hand, Koechlin et al. (2021) further emphasized the significant placebo response observed for depression [24].

Regarding the overall QOL, our finding of a small but statistically significant improvement is based on high-quality evidence. Walitt et al. (2015) similarly found SSRIs to be statistically and clinically superior to placebo for disease-specific QOL, with a moderate effect size, while also reporting an appreciable clinical benefit for global improvement [5]. Häuser et al. (2012) and Häuser et al. (2009) both reported significant, small effect sizes for QOL (health-related quality of life (HRQOL)) with SSRIs [6,22]. Üçeyler et al. (2008) noted that 75% of studies reported QOL improvement with SSRIs [25], and O'Malley et al. (2000) associated SSRIs with improved overall well-being [23].

Reflecting the inherent inconsistencies in the literature, Choy et al. (2011) found no significant difference between SSRIs and placebo for any outcome due to a lack of suitable data and generally poorer quality studies [26]. Üçeyler et al. (2008) also noted that studies with low methodological quality reported more positive outcomes [25]. Moreover, Koechlin et al. (2021) suggested that treatment with SSRIs alone may "fall short" in the treatment of fibromyalgia [24].

Explanation

The body of evidence underscores that while SSRIs demonstrate statistically significant benefits across certain fibromyalgia symptoms like pain, depression, and QOL, the magnitude of these effects can be modest, and consistency across studies varies, particularly for direct pain measures. The presence of some studies showing clear benefits (e.g., Arnold et al., 2002; Patkar et al., 2007 for FIQ) [7,19], others showing mixed results (e.g., Arnold et al., 2002 for pain versus depression) [19], and some demonstrating no effect (e.g., Nørregaard et al., 1995; Wolfe et al., 1994) [15,20] for overall outcomes, contributes to the considerable heterogeneity observed in our meta-analysis. Methodological differences, such as blinding, sample size, duration, specific SSRI and dosage, and the handling of concomitant medications, appear to be crucial factors in these conflicting outcomes.

The overall quality of the evidence for SSRI efficacy in fibromyalgia remains compromised by significant limitations in the included studies. The quality of evidence was rated as low for most outcomes, including pain and depression, primarily due to substantial heterogeneity and a high risk of bias identified across the studies, which corroborates the findings of Walitt et al. (2015) and Häuser et al. (2012) [5,6].

Limitations

While our study highlights significant benefits in terms of pain, depression, and QOL, there are qualifications regarding the quality of evidence that acknowledge the noted limitations in the broader literature. 

Several issues raise questions about the internal validity of primary studies. For one, randomization and allocation concealment were unclear or inadequately described in about half of the studies [15,18,20,21], which may have introduced selection bias. Furthermore, performance and detection bias were identified in six studies due to inadequate blinding procedures. Four studies raised some concerns related to issues such as unclear blinding protocols [7,20], the inability to implement double-blinding [16], or deviation related to the intervention [17]. Two additional studies were judged to be at high risk due to the absence of prober binding methods [18,21]. The high attrition rate (particularly in the placebo group) contributes to missing outcome data, which may have led to an overestimation of the treatment effect of SSRIs. Other concerns include inappropriate analysis or inconsistently applied ITT analysis, as well as potential reporting bias.

Furthermore, the external validity of primary studies is limited by the substantial heterogeneity observed across trials, particularly in outcomes related to pain and depression. This reduced confidence in the pooled effect estimates suggests that the overall results may not be generalizable to all patient populations or clinical settings. Factors contributing to this heterogeneity likely include variations in sample sizes, study durations, SSRI type and dosage, demographic representation, and the absence of subgroup analysis (forest plot analyses in Figures 2-4).

The limitations of the present meta-analysis are mainly evident in the specific criteria used for including and excluding studies, which, while necessary for a focused review, impose limitations on the generalizability and comprehensiveness of our findings. We only included RCTs published in peer-reviewed journals. Thus, other potentially valuable forms of evidence were not considered. While clinical practice often employs the use of multiple drugs, studies that would have otherwise compared the isolated effect of SSRIs to non-SSRI antidepressants (e.g., SNRIs, TCAs, MAOIs) were not included, aside from a single study [14]. This omission limits our ability to assess the potential benefits of combination therapies and other antidepressants that are commonly used in treating fibromyalgia. Furthermore, because only studies with a control group were eligible, and those that did not report pain or depression outcomes as primary or secondary measures were excluded, this may overlook studies that explored other important aspects of fibromyalgia management.

Recommendations

We recommend that future RCTs apply a rigorous methodology to reduce the risk of bias. This can be accomplished by improving randomization, implementing effective blinding procedures, minimizing incomplete outcome data, employing appropriate analysis, ensuring transparency, and preventing selective reporting. 

In order to address heterogeneity, studies should aim to utilize a more consistent set of validated and widely accepted outcome measures for pain and depressive symptoms with a clear scale. Researchers should consider conducting studies designed to explore potential sources of variability, such as different SSRI types and dosages, patient characteristics, and the duration of the study.

The reviewed studies had varying durations, ranging from as short as six to eight weeks. Fibromyalgia is a chronic disorder; therefore, understanding long-term adherence and effectiveness in real-world settings is crucial. We recommend long-term studies to assess the sustained efficacy and safety of SSRIs beyond the short to medium term. Moreover, subgroup analyses or individual participant data meta-analyses could be valuable in understanding which patients are most likely to respond to SSRIs. In order to better understand the distinct effects of SSRIs on pain and mood symptoms, future studies could specifically target fibromyalgia patients with and without comorbid major depressive disorder.

While this review focused exclusively on SSRIs, fibromyalgia management often involves other antidepressant classes like SNRIs or TCAs, and sometimes combination pharmacologic agents. Future research should directly compare the efficacy and safety of SSRIs with other commonly used antidepressants or investigate the role of SSRIs in combination therapies where an isolated analysis of the SSRI effect can be discerned.

## Conclusions

We performed a high-quality systematic review and meta-analysis assessing the efficacy of SSRIs in treating chronic pain and comorbid depression among individuals with fibromyalgia. While SSRI interventions demonstrated statistically significant benefits in reducing pain and depression symptoms and modestly improved disease-specific QOL compared to placebo, the overall quality of evidence was frequently low or very low, largely due to substantial methodological heterogeneity and identified risks of bias across the included studies. We recommend that future RCTs adopt more rigorous methodologies to minimize bias, thereby significantly enhancing the overall quality of evidence and strengthening the existing literature.
